# Characterization of Noise Level Inside a Vehicle under Different Conditions

**DOI:** 10.3390/s20092471

**Published:** 2020-04-27

**Authors:** Daniel Flor, Danilo Pena, Luan Pena, Vicente A. de Sousa, Allan Martins

**Affiliations:** 1 Department of Communications Engineering, Federal University of Rio Grande do Norte, Natal 59078-970, Brazil; 2 Department of Electrical Engineering, Federal University of Rio Grande do Norte, Natal 59078-970, Brazil

**Keywords:** noise sources, regression analysis, contribution analysis, vehicle interior noise

## Abstract

Vehicular acoustic noise evaluations are a concern of researchers due to health and comfort effects on humans and are fundamental for anyone interested in mitigating audio noise. This paper focuses on the evaluation of the noise level inside a vehicle by using statistical tools. First, an experimental setup was developed with microphones and a microcomputer located strategically on the car’s panel, and measurements were carried out with different conditions such as car window position, rain, traffic, and car speed. Regression analysis was performed to evaluate the similarity of the noise level from those conditions. Thus, we were able to discuss the relevance of the variables that contribute to the noise level inside a car. Finally, our results revealed that the car speed is strongly correlated to interior noise levels, suggesting the most relevant noise sources are in the vehicle itself.

## 1. Introduction

Acoustic noise has been considered a crucial issue and one of the most important topics in sensing and communication systems in vehicles in the last years. General vehicle applications depend on the audio signal quality such as multimedia [1,2,3,4], security [5,6,7,8,9], and assistive [10,11,12] and autonomous vehicle applications [13,14]. In addition, vehicle noise is a concern to researchers due to its effects on human health and comfort, both inside and outside the vehicle. The influence of environmental noise in sleep and mental health [15,16,17] has been investigated. There is also research on the impact of noisy environments on the performance of students in school [18] and on the development of the cognitive processes in children [19]. Exposure to road and transportation related noise has been associated with a higher risk of ischemic heart disease [20], myocardial infarction [21], and diabetes [22]. These topics have motivated studies on better modeling and evaluation tools for acoustic noise with machine-learning based approaches [23,24]. Notably, a study uses an artificial neural network technique to model the sound quality of vehicle interior noise [25] and another one proposes a new sound quality metric for vehicle suspension shock absorber noise [26].

These interior noises result from a composition of different noise natures, such as wind noise, engine noise, and rolling noise. The understanding of the more relevant noise sources might indicate what are the key challenges in acoustic systems and what are the better mathematical models for them. Therefore, their evaluations are fundamental for anyone who is interested in vehicular acoustic signal analysis.

There are different noise source contributions to car environments and they can be separated into many different aspects. According to the literature [27], vehicle interior noise can be focused on vehicle subsystems and components, such as tire-road interaction noise [28,29] or aerodynamic noise. The tire-road interaction is usually refereed by two components, namely structure borne noise, contributing to low-frequency excitation (below 500 Hz), and air borne component with mid- and high-frequency excitation (above 500 Hz). Understanding the characteristics of this particular source of noise is essential in the development of low-noise road surfaces [30,31,32]. It has also been investigated the sound quality related to specifics noise phenomena in vehicles, such as closing doors [33,34], engine sound [35,36], and wind noise [37,38]. The authors of [39] presented the frequency characteristics and a model for wet road traffic noise, while the authors of [40,41] proposed wet road detection schemes based on acoustic measurements. However, to our knowledge, no efforts have been made to evaluate the effects of rain on noise inside the vehicle.

In the evaluations, the efforts are focused on finding better contributions to represent the general sound quality from interior and exterior vehicle noises. However, although many works show us the effect of different contributions to the acoustic noise in vehicles, little attention has been paid to statistical analysis of individual noise sources. For example, the authors of [42] showed the contribution of air-conditioner noise in sound quality analysis and more recently, the work in [43] establishes correlations between some features and the acoustic noise in cars.

One way to evaluate the acoustic noise contributions inside cars is to measure acoustic signals using microphones in real conditions. For this approach, a set of controlled or known relevant conditions is specified, and the other uncontrolled or unknown noise sources are spread in invariant random events, which ensure that these kinds of noise sources will not cause a disturbance in the analysis such as a bias. In this case, the uncontrolled or unknown variables will interfere with the same frequency that they happen in a real scenario. As a result, this experimental evaluation allowed us to investigate the degree of influence of sources into acoustic noise inside a car. Based on these criteria, the specifications were defined carefully considering the most relevant controlled noise sources in the experiment.

Even though studies considering all vehicle noise sources have many advantages, they have not been developed due to the amount of resources (time and cost) necessary in the analysis of the entire vehicle system. On the other hand, a reduced analysis of the main noise sources contributions in a vehicle makes the study more feasible and realistic. Thus, the variables with higher contributions to noise levels suggest where researchers should focus on when designing noise mitigation systems, such as filters and acoustic noise control.

Thus, one of the objectives of this study was to statistically analyze 212 acoustic noise measurements conducted on different known conditions. The procedure is described in two parts: measurements and evaluations. First, we planned the measures using the controlled and uncontrolled variables. The known or controlled variables were defined based on preliminary experiments, in which we evaluated their main contributions qualitatively. After that, we established the criteria, relationship between the variables, and the constraints. Then, we measured and checked the consistency of the data in relation to the variables. Finally, we evaluated the data using statistical tools such as linear regression and Pearson correlation among the variables and the power noise levels.

The present paper examines possible noise sources correlated with noise levels in an attempt to help researchers who study how to reduce noise levels and improve sound quality in the vehicle interior.

In this paper, our key contributions are:

- Acoustic measurements were collected in several conditions (weather, car windows position, car speed, and traffic level).
- The data collected herein, including information on the conditions and location of each measurement, are freely available [44] and can help researchers in different purposes.
- Statistical evaluation of the different conditions in relation to noise levels was performed.

This paper is organized as follows. In Section 2, we describe all known and controlled variables and measurement conditions, presenting the process of select and organize the data. The measurement setup is described in Section 3. In Section 4, the main source contributions are evaluated and discussed quantitatively and qualitatively. In Section 5, we present our final remarks and further investigations.

## 2. Methods

### 2.1. Environment Variables

Natal is a city located in northeastern Brazil, and has a population of about 900,000 and an area of 167 km${}^{2}$, considered the second smallest capital of Brazil. Natal has a typical tropical climate, with warm temperatures and high humidity throughout the year. The average low and high annual temperatures are 23 ${}^{\circ }$C ($73^{\circ }$F) and 29.7 ${}^{\circ }$C ($85.5^{\circ }$F), respectively, and the average annual precipitation in the year is 1721.4 mm (67.77 inches). The measurements were carried out in June and July, the coolest months with an average low temperature of 22 ${}^{\circ }$C ($71^{\circ }$F) and an average high temperature of 29 ${}^{\circ }$C ($84^{\circ }$F).

**Figure 1:**
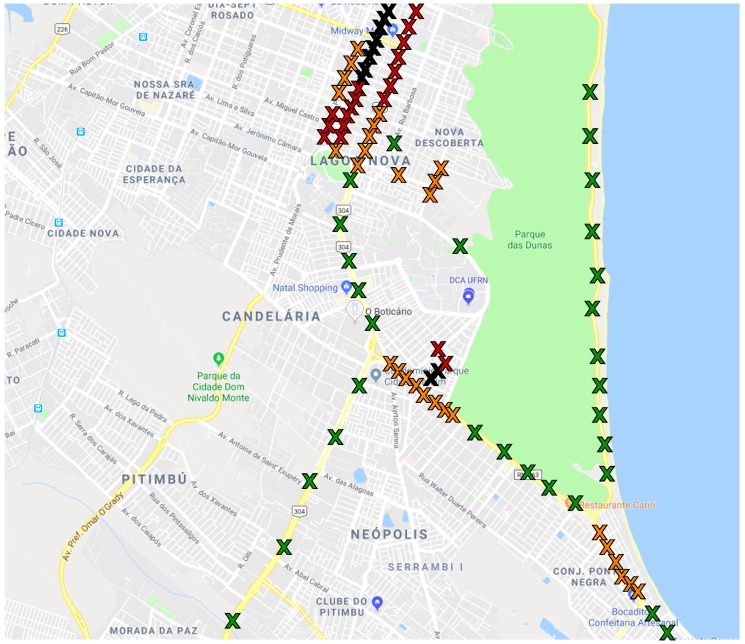
Map of Natal with traffic signs.

The sampling points were located in different streets and avenues spreading the uncontrolled conditions such as crowd and traffic, as illustrated in Figure 1. The traffic conditions were defined following the Google Maps traffic conditions policy [45]. Each sampling point was assigned to one of four possible traffic conditions in a specific location. Those locations may have different features, which is why the sampling point was spread for different regions of the city. For example, while the highway near the coast (with 6.2 m) has strong winds blowing from the ocean that may cause higher noise levels, the quiet streets usually present lower noise levels. All measurements were obtained on asphalt with smooth road surface conditions with no presence of potholes or unevenness.

Table 1 presents all the possible conditions of the four environmental variables that were controlled during the measurements. Those are the position of the car windows, the presence of rain, the traffic condition, and the maximum speed of the car.

We acquired a large number of measurements. Care was taken to obtain data for the combination of all possible conditions of the controlled variables. During a measurement, the participants did not speak or make any noise. To identify outliers in the data, we reviewed the audio signals to check for highly impulsive events (such as sounds due to potholes in the road or a person’s sudden shouting near the vehicle).

For measurements with no rain, all four windows were either fully open or closed. In the case of measuring during rainfall, the windows were kept closed.

We also measured noise levels in different traffic conditions, as shown in Table 2. To record this information, we utilized Google Maps’ color codes for traffic and noted the color of the road displayed on the application during measurement. For example, when measuring in a high-speed highway with no traffic delays, the condition was recorded as Green.

Finally, we also recorded the maximum speed of the car during the interval of each measurement. The speed of the car was always compatible with the traffic condition displayed in Google Maps. Table 2 shows the speed intervals for each traffic category.

### 2.2. Statistical Methods

Our goal was to understand how each environmental factor affects the noise level inside a vehicle. To achieve this, we utilized visualization tools such as histograms and box plots to analyze the data. We also employed statistical modeling to highlight the relationship between the studied variables.

Initially, we quantified the signal power for each measurement. There are many different ways to calculate signal energy or power. One approach is to compute the energy from the cepstral coefficients [46]. The cepstral coefficients are a set of features obtained by first taking the natural logarithm of the magnitude of the Fourier transform of a signal, and then obtaining the inverse Fourier transform of the result. They are often applied in speech recognition and transcription tasks. Another approach is to use the Teager–Kaiser (TK) operator. The TK operator is a measure of energy that takes into account both the signal’s amplitude and frequency. Despite their low complexity, the operators and their derivations are capable of estimating useful features of a signal such as instantaneous frequency and spatial envelope and phase [47]. They can be used, for example, in the instantaneous estimation of AM-FM signals and images. For our objective in this work, however, it was sufficient to compute the average power of the measurements in the following way:

(1) $$P_{dB}=20\;\cdot \;\log\left(\frac{1}{N}\sum_{n=0}^{N-1}x^{2}\left(n\right)\right),$$

where *N* is the length of sampling and x(n) is the voltage signal from the microphone.

The dataset contains 212 samples, with five features for each measurement: noise power, presence of rain, window position, traffic condition, and maximum speed. Power and speed are numeric, while the three other are categorical. We encoded the latter using natural numbers. The binary variables (window position and rain) were encoded with 0s and 1s. Traffic condition was encoded in descending order of severeness, i.e., “Green” corresponds to 3, and “Black” corresponds to 0.

We then performed an initial exploratory analysis. For the numeric features, we obtained a histogram plot to understand the distribution of power and speed data. We also obtained a histogram for noise power level by traffic condition to compare the distribution of noise level for each condition. For categorical data, we obtained the box plot of power levels for each category separately to highlight the difference of noise levels in them.

Next, we created a linear regression model for each feature. In the models, power is always the explanatory (dependent) variable and the other features are the response (independent) variable. The linear regression model, based on second-order, is the simplest feature extractor. It can be used to measure to what extent two or more variables have a linear relationship. Even if this relationship is only approximately linear, the model is a simple way to identify the influence of the inputs in the model output. To compute the models (estimate the coefficients of a linear regression model), we used the Ordinary Least Square (OLS) method [48]. It does so by minimizing the sum of the squared differences (residuals) between the observed dependent variable and the prediction line.

We computed three metrics of the goodness of fit to compare the influence of the environment features in the average noise power. First, we obtained the mean squared error (MSE) [49]. The MSE is the average of the square of the errors between the model and the actual values. A smaller MSE indicates a better fit, although the actual values of MSE depend on the scale of the data. It is mostly used to compare different models for the same response variable in the same scale. We also computed the coefficient of determination, R^2^ [49]. This value is the ratio of the sum of squared residuals to the variance of the actual data values. It is always between 0 and 1 and represents the variation in the response variable that is accounted or explained by the model. In the context of acoustic noise, the R^2^ is also related to the noise power, that is, how much of the noise power can be attributed to the explanatory variables.

The R^2^ can highlight the correlation between variables. However, it is not a complete description of the goodness of fit of a model. The R^2^ assumes that all independent variables in the model explain the variation in the response variable. It always increases when more variables are added to a model, even if they in reality do not affect the independent variable. Thus, it does not evaluate the significance of the relationships shown by the model [49].

A way to verify this significance is by computing the F-statistic [49]. Similar to the R^2^, the F-statistic (or F-value) compares the explained and unexplained variation in the model, but weighted by the degrees of freedom of the model, that is, how many model coefficients are used in relation to the number of observations. Thus, it takes into account the complexity of the model.

The F-statistic is used in the F-test. In this test, the null hypothesis is that the model coefficients are zero, and the alternative hypothesis is that at least one coefficient is not zero. The F-test shows if the relationship between the variables is a result of chance or not. The higher is the F-value, the more significant are the results drawn from the model.

For categorical data, the linear regression model can be used to describe the relationship between two of more variables. However, it is not always adequate to represent this relationship as a linear function, as there is a limited, discrete range of values for the response variable. Thus, we also obtained a logistic regression model for the binary variables [49]. The logistic model was obtained by transforming the predicted values of the linear model to another scale that is bounded by 0 and 1. Thus, the output of the model can be interpreted as a probability that a data point belongs to a certain category, and the coefficients of the model are adjusted to find the best match of these probabilities to the data. For logistic models, the goodness of fit metrics described above are not used. To compare the models, we computed McFadden’s Pseudo-R^2^ [50]. While its calculation is different from the regular R^2^, it has a similar interpretation.

We concluded our analysis by measuring the relationship between the environmental variables, highlighting how much correlation they present with each other. We also built a multiple variable regression model, using speed as the dependent variable and the reminder as explanatory variables. We compared the contribution of each variable, and how much better a model with multiple predictors is than the previous one variable model.

## 3. Measurement Setup

**Figure 2:**
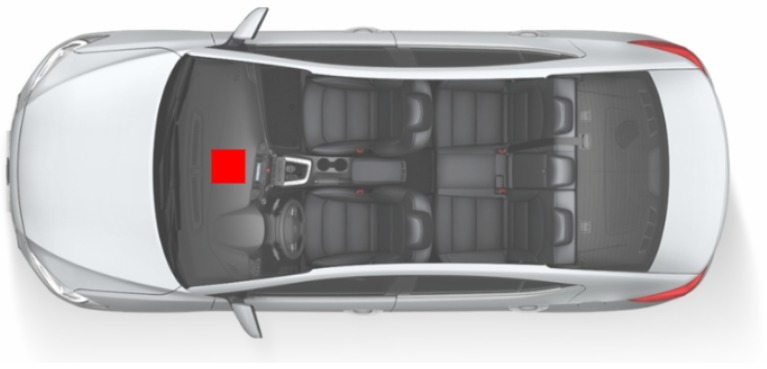
Top view of the car cabin showing the microphone as a red square [52].

We selected a sedan C4 Lounge from Citroen with automatic transmission as the vehicle for our measurements. The measurement setup used is similar to the one presented in [51]. It consists of a ReSpeaker Core v1 (MT7688) board using the Analog-to-Digital Converter (ADC) AC108 with four ADC delta-sigma, with four microphones connected to a Raspberry Pi 3 (model B) processor to collect, compute the average power, and store the data. In our previous work [51], this setup was validated with a Data Acquisition (DAQ) NI-6361 from National Instruments. It was positioned on the panel, inside the cabin, similar to the position of the multimedia microphone, as illustrated in Figure 2.

**Figure 3:**
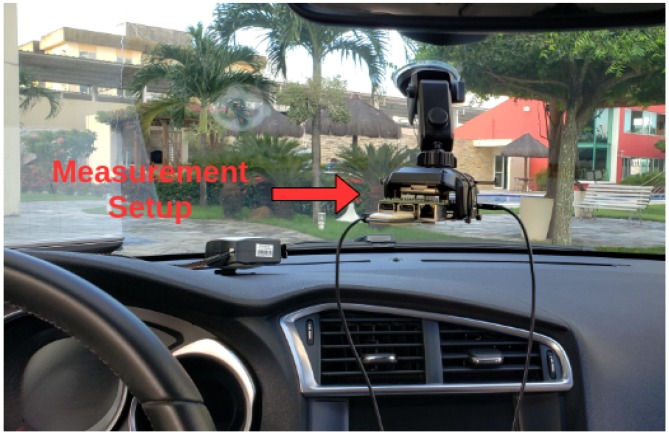
The measurement setup.

Figure 3 shows the measurement setup suspended on the panel stuck on the windshield by a stand. The data were measured using the ReSpeaker at 48 kHz of sample rate. In total, 240,000 samples are collected in 5 s, from which the average power was computed.

The measurements were classified based on the known categorical parameters divided into 12 conditions and their combinations representing door window, rain, speed, and traffic conditions. For each measurement, all parameters and observations were manually recorded in a diary, and the power noise levels were computed in the Raspberry Pi. Moreover, we collected the spatial position, aiming to spread the observations regarding the unknown parameters making them independent.

## 4. Results and Discussions

### 4.1. Measurements Presentation

Table 3 presents the number of measurements for each condition of the controlled environmental variables. The number of samples is balanced between the conditions, except for the “Presence of Rain” variable, which contains a significantly higher number of measurements with no rain because of the weather conditions in northeastern Brazil. The table also shows the encoding information of each feature.

**Figure 4:**
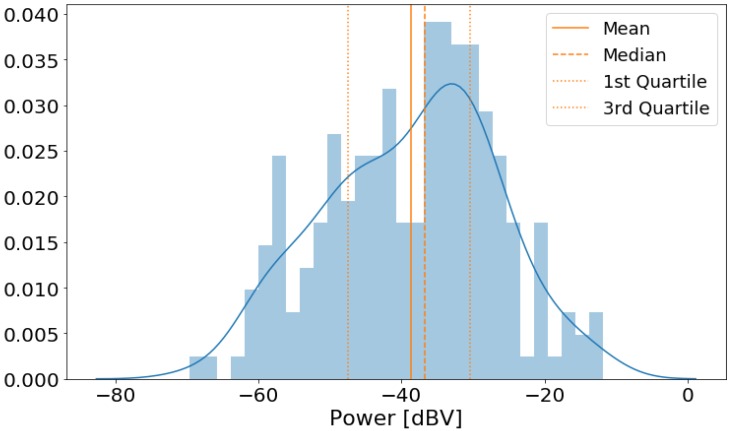
Histogram of measured noise power levels.

Figure 4 shows the distribution of the noise power levels in dBV, along with some descriptive statistics. The histogram has a bell-like shape, with 50% of the measurements between −47.58 dBV and −30.35 dBV.

### 4.2. Traffic Analysis

**Figure 5:**
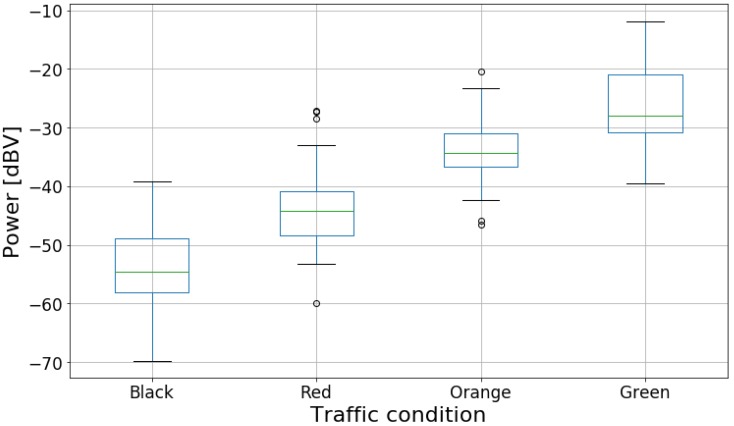
Box plot of noise power data divided by traffic categories.

**Figure 6:**
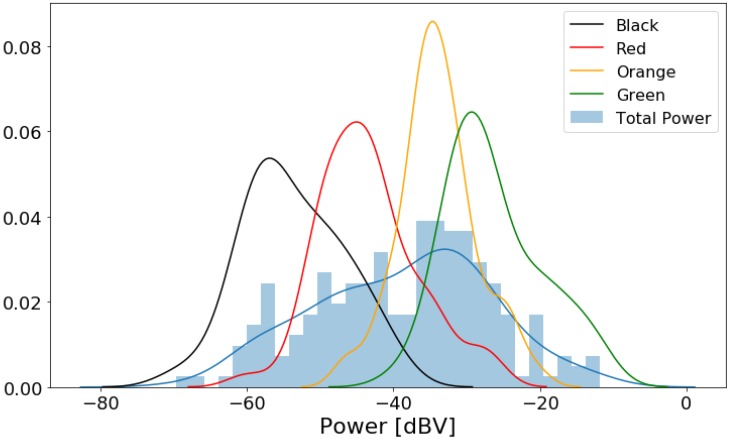
Histogram of noise power data with distribution curves for each traffic category.

Figure 5 presents the box plot of the power data grouped by traffic conditions. The box plots have an ascending order from “Black” to “Green”, showing that as traffic becomes less severe, the noise power level in the car tends to increase. Figure 6 shows another visualization of the noise power level distribution. Analyzing the figure, the modes for each category are separate despite the significant overlap between the curves.

These results suggest there is some correlation between noise power and traffic, and, by association, noise power and the speed of the car. To evaluate this relationship, we built a linear regression model in the form

(2) $$\mathrm{Traffic}\approx a_{0}+a_{1}\;\cdot \;\mathrm{power}\;,$$

where $a_{0}$ is the intercept and $a_{1}$ is the coefficient of the explanatory variable (power level). Even though the response variable is categorical, we chose to fit a linear model due to the ordered nature of the traffic data, as well as due to the trend implied in Figure 5 and Figure 6.

**Figure 7:**
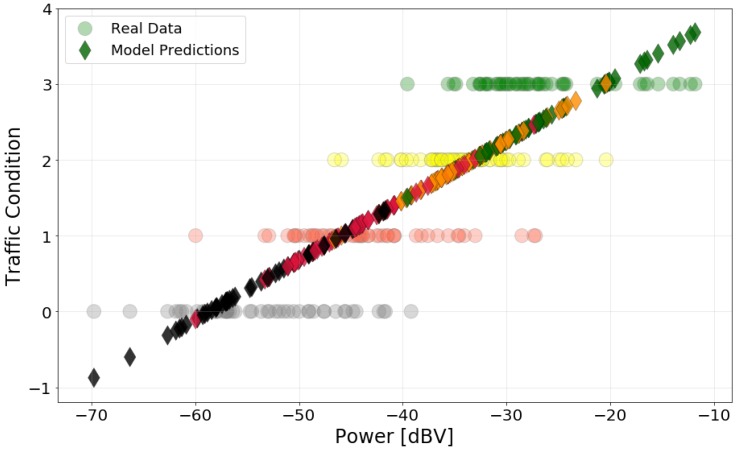
Traffic data and predictions using the linear model. Colors represent the real traffic category of the predictions.

The model obtained is presented in Figure 7. The circles are the actual data points, and the diamonds are the predictions. The colors represent the actual traffic category of data points and predictions. The model shows that higher power level implies a better traffic condition, which agrees with the behavior displayed by the box plot. Visually, one can see that the predictions are centered around their actual values of traffic, although there is some variation that causes overlap between the categories. For example, the red diamonds are centered around Traffic=1.

The goodness of fit metrics for the model are presented in Table 4. It also presents the coefficients of the model and their 95% confidence interval. The R^2^ value indicates that 71.27% of the variance in Traffic is accounted for by the model. This implies a strong relation between the variables. The significance of this relationship is confirmed by the high F-value and its low probability. The MSE presents a low value; however, this is due to the categorical nature and small scale of the traffic data (from 0 to 3). Therefore, the MSE does not provide much information about the quality of the model in this case.

### 4.3. Rain Analysis

**Figure 8:**
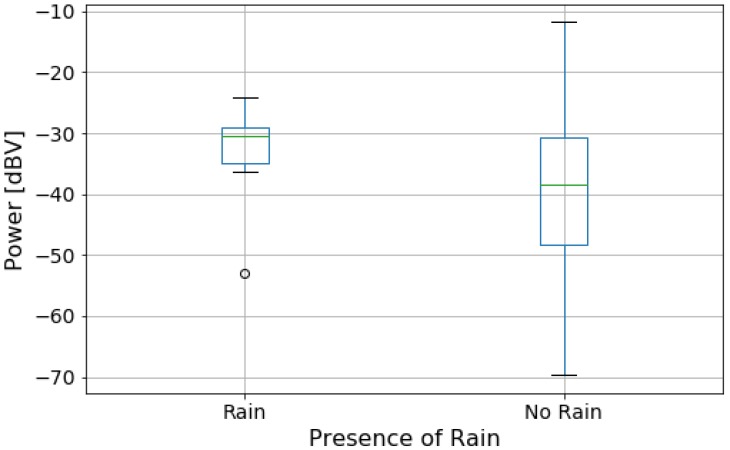
Box plot of data divided by presence of rain.

Figure 8 presents the box plot of power data grouped by the presence of rain. Contrary to the previous case, the box plots for this variable have very distinct shapes. The “No Rain” category presents a much bigger variation in noise than the other case and its relation to noise levels are not intuitive. This behavior might be explained by the low number of measurements. Although we have only 18 points (Table 2), and those points may not be enough to represent this category statistically, these findings suggest a model where an external factor can contribute to the noise levels and do not have any relation to the position of the measured location, but represent an environmental parameter. This study, therefore, suggests that non-traditional factors can affect the noise level and they can even produce unexpected results. Most notably, this is the first study to our knowledge to investigate the rain contribution to the noise level measured in the setup located on the car panel.

**Figure 9:**
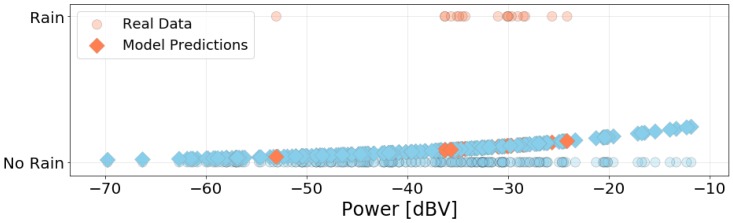
Data grouped by presence of rain and predictions using the logistic model.

Due to the distribution of data by category presented in Figure 8, it is expected that both the linear and the logistic models will fit poorly to the data. Nonetheless, we built those two models to evaluate the relationship between power and presence of rain, and also to provide a base of comparison with the next variable (window position). The linear model is in the form

(3) $$\mathrm{Rain}\approx b_{0}+b_{1}\;\cdot \;\mathrm{power}\;,$$

where $b_{0}$ is the intercept and $b_{1}$ is the coefficient of the explanatory variable (power level). Table 5 and Table 6 present the goodness of fit metrics for this model. Figure 9 presents the logistic model predictions. The circles are the actual data points, and the diamonds are the predictions. From the small R^2^, F-Statistic, and Pseudo-R^2^, the model shows no significant relationship between noise power and rainfall. Our data have more measurements for one category and, as shown in Figure 8, the noise power levels in the “Rain” scenario are completely contained in the range of values for the “No Rain” scenario. As presented in Figure 9, the model predicts all data points as belonging to the “No Rain” group. Thus, this model provides no information about whether it is raining or not based on noise power acquired inside the car. However, more data must be collected to create a more representative model of the raining scenario. This is a matter of our further studies, especially considering the shortage of rainfall at the measurement site.

### 4.4. Car Windows Analysis

**Figure 10:**
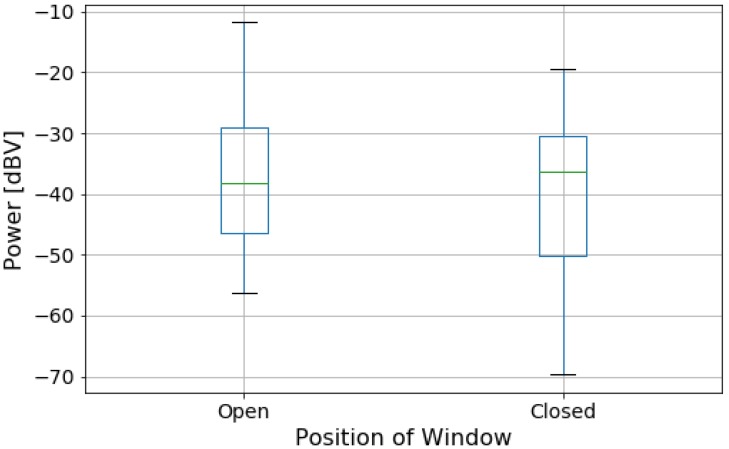
Box plot of data divided by the state of the car windows.

**Figure 11:**
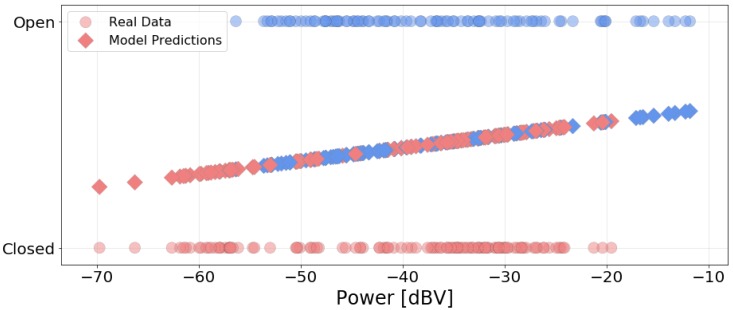
Data grouped by position of windows and predictions using the logistic model.

Figure 10 presents the box plot of power data grouped by the position of the car windows. From the position of the plots, the noise power levels tends to be higher when the windows are open, as this allow for more external noise in the car. Unlike the previous variable, the shape of the box plots are similar, that is, the range of values is similar regardless of the condition. However, there is significant overlap between the two blox plots: only 8.45% of the measurements in the “Open” group have a power level above the maximum power level in the “Closed” group. This indicates that the linear and logistic models will not be able to represent the data, similar to the Rain variable, as there is not enough distinction in noise power between the two conditions. The linear model is in the form

(4) $$\mathrm{Window}\approx c_{0}+c_{1}\;\cdot \;\mathrm{power}\;,$$

where $c_{0}$ is the intercept and $c_{1}$ is the coefficient of the explanatory variable (power level). Table 7 and Table 8 present the goodness of fit metrics for the models, while Figure 11 shows the resulting logistic model. Visually, we see the poor distinction between the categories. Table 7 shows that, while the R^2^ and F-value are slightly bigger for the window case (compared to the Rain analysis), both sets of models perform poorly in regards to the metrics and fail to represent the data. This implies a weak relationship between their response variables (rain and window position) and noise power.

### 4.5. Speed Analysis

**Figure 12:**
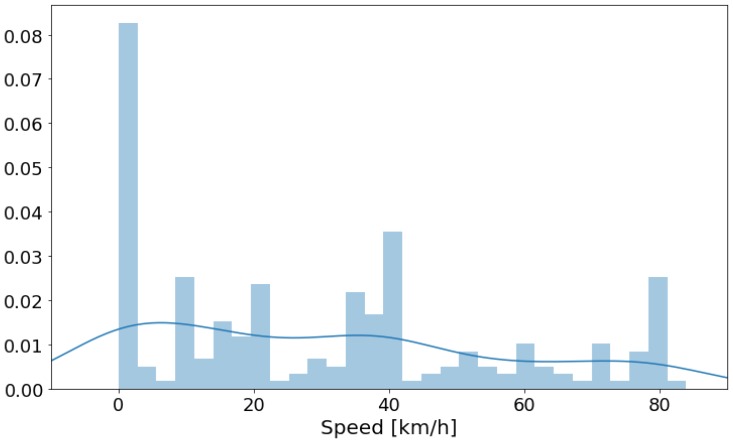
Histogram of the maximum speed of the car during the measurements.

Figure 12 presents the speed data of the measurements in a histogram. There is a higher number of measurements for the speed of zero. Those data points correspond to the “Black” traffic category, when the car is stationary due to a heavy traffic jam. As shown in Table 3, the numbers of measurements are balanced between traffic categories. Thus, the speed data are also balanced in accordance to the traffic categories.

**Figure 13:**
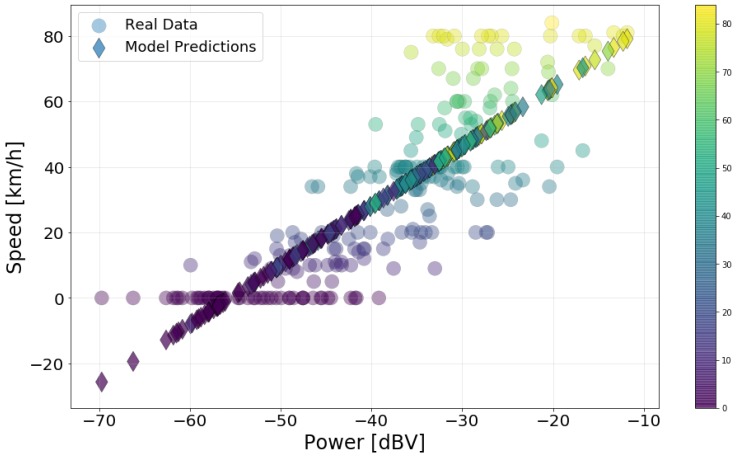
Speed data and predictions using the linear model.

Of all environmental variables, speed is the only one numeric in nature. Therefore, we obtained a linear regression model in the form:

(5) $$\mathrm{Speed}\approx d_{0}+d_{1}\;\cdot \;\mathrm{power}\;,$$

where $d_{0}$ is the intercept and $d_{1}$ is the coefficient of the explanatory variable (power level). Figure 13 presents the resulting model predictions and the actual data. They show that a higher car speed implies a higher noise level inside the car, a result similar to that presented in Figure 7. As above, the circles are the actual data points, and the diamonds are the predictions.

Table 9, which lists the goodness of fit metrics, reaffirms the result that the model is a good representation of the data. The R^2^ value indicates that roughly 67% of the variation in speed is explained by the model. The F-value is also high, indicating a significant relationship. These results, compared with the one for the window variable, indicates that there is more contribution to the interior noise levels from the vehicle itself than the wind [27].

The MSE value may seem high, specially compared to the three previous models. However, this comparison is not relevant as MSE is not an adequate metric for categorical data. Furthermore, the scale of speed data is bigger than that of the other variables, resulting in higher error values on average. Finally, the simple linear regression cannot model the fact that speed data cannot be negative. Since there is a high number of zero data points, a high MSE is expected.

### 4.6. Multiple Variable Analysis

**Figure 14:**
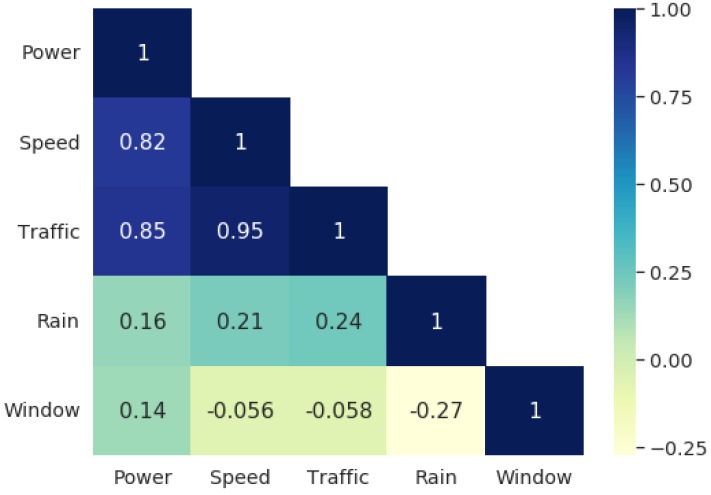
Correlation matrix of the dataset.

The previous analysis indicated the speed of the car and traffic conditions contribute the most to the noise power inside the car, while the position of the windows and rain presented a weak influence. Another way to verify how strong is the relationship between the variables and noise power is by computing their cross-correlation. Figure 14 presents a visualization of the correlation matrix of the data. Noise power has a high correlation with both speed and traffic, and a low correlation with the state of the windows. This confirms the behavior presented in Figure 7 and Figure 13 that noise power tends to increase with the speed of the car. It also reaffirms the result presented in Figure 11 and Table 8 that the window variable has low explanatory value in the model.

Figure 14 also shows a correlation of 0.94 between traffic and speed. This high correlation is expected. As stated in Section 2.1, the traffic categories were obtained in Google Maps by averaging the speed of the cars reported by the application’s users. The correlation is not exactly 1 due to variations in driving speed during measurement for each traffic scenario. Nonetheless, in the context of statistical modelling, traffic and speed convey roughly the same information about the response variable and can be considered redundant.

To illustrate this redundancy between speed and traffic, we built a model to predict the speed of the car using all the other variables as independent.

(6) $$\mathrm{speed}\approx e_{0}+e_{1}\;\cdot \;\mathrm{power}+e_{2}\;\cdot \;{\mathrm{traffic}}_{\mathrm{red}}+e_{3}\;\cdot \;{\mathrm{traffic}}_{\mathrm{orange}}+e_{4}\;\cdot \;{\mathrm{traffic}}_{\mathrm{green}}+e_{5}\;\cdot \;{\mathrm{rain}}_{\mathrm{yes}}+e_{6}\;\cdot \;{\mathrm{window}}_{\mathrm{open}}\;,$$

where $e_{0}$ is the intercept and $e_{1}$ is the coefficient of the power level; $e_{2}$, $e_{3}$, and $e_{4}$ are the coefficient added when traffic conditions are red, orange and green, respectively; $e_{5}$ is the coefficient added when there is rain in the sample; and $e_{6}$ is the coefficient added when the windows are open. Although we do not expect a physical relationship between the rain and window variables and speed, we include these variables in this model to verify that they do not influence in the results. In order words, we want to verify that there is no bias in the measured speed data in relation to the absence or not of rain and the state of the car windows.

**Figure 15:**
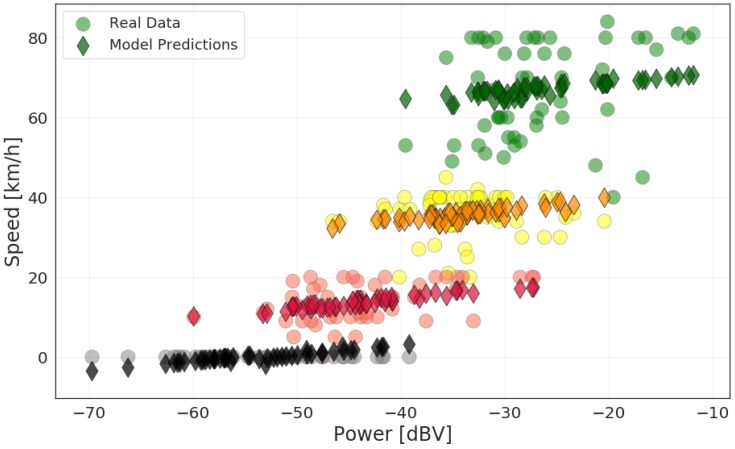
Speed data grouped by traffic conditions and predictions using the linear mode with all explanatory variables.

The model has prior-knowledge about the speed interval during measurement, which is conveyed by the traffic variable. The results shown in Figure 15 illustrate this. There are four groups of predictions divided by the traffic categories. No prediction is grouped incorrectly. Each traffic group has four lines, corresponding to the possible combinations of window and rain variables. These lines lie close to each other, indicating that the models for each pair of those conditions give similar predictions. This is expected, as speed has no relationship with rain and window, and the speed measurements were collected in a balanced quantity for all possible conditions of the variables. Effectively, the variable power, which determines the slope of all lines, is the one that determines the speed in each traffic group.

The goodness of fit metrics of the model are presented in Table 10. The high R^2^ value indicates that most of the variation in speed is accounted for by the model. However, comparing Table 9 (noise power as the only explanatory variable) and Table 10, there is not much improvement in the F-value with the addition of the three other variables. Thus, the traffic variable does not contribute much to the model of speed, due to its redundancy. We conclude that either traffic or speed can be used as a good explanatory variable to noise power inside a vehicle, but not simultaneously.

## 5. Conclusions

Acoustic noise is a central issue in vehicle design. It is expected that will gain more attention as health concerns and multimedia, security, and autonomous vehicle applications become more prevalent. Prior work has shown different contributions to noise levels in the vehicle interior, investigating its subsystems and components. Those studies are mostly conducted in laboratory environments or using mathematical models. However, they may underestimate or ignore noise sources from specific conditions inside or outside the car. In this study, we presented an experimental evaluation of the contribution of different acoustic noise sources inside a car. The experiments were carried out by using a low-cost measurement setup inside a vehicle to acquire noise power levels in different traffic areas and different controlled conditions. Data visualization, statistical modeling, and goodness of fit metrics were used to assess the influence of speed, traffic, rain, and position of the car windows.

Our experiments in real traffic conditions showed a strong correlation between the speed of a car and its interior noise level, likely due to higher noise generation in the motor at higher speeds. Those results are correspondent with our general theoretical assumption. In contrast, the state of the car windows seems to not contribute significantly to the measured noise. The same, even with few collected data, can be speculated about the presence of rain. This could imply that most of the noise inside the vehicle can be attributed to its operation and movement, creating a higher variation on noise level and thus reducing the correlation of these less dominant factors. Thus, the results suggest that efforts to improve acoustic quality inside a vehicle should be focused on reducing the noise generated by the car itself.

To further our research on this topic, we plan to collect more noise data and study more variables, such as wind speed and different vehicles, as well as considering the noise in different car positions. We also plan to evaluate the vehicular scenario with the presence of human speech sources in the driver’s seat, passenger’s seat, and backseat. We also plan to investigate the spectral characteristics of the measured noise, which is especially important for noise suppression purposes. The data collected could then be explored in machine learning tasks and source location problems in the context of vehicle applications. In addition, more data may be acquired in rain conditions, enriching our study by providing more data points at this condition. Finally, an investigation of the impulsiveness of acoustic noise [51] in the vehicular scenario is warranted.

## Figures and Tables

**Table 1:** Conditions of the controlled environment variables.

Variable	Possible Conditions	Constraints
Window Positions	Open; Closed	Open: only with no rain.
Rain	Yes; No	Yes: only with closed window.
Traffic	Black; Red; Orange; Green	Each traffic has a range of speed (see Table 2).
Speed	0–80 km/h	-

**Table 2:** Conditions of the traffic.

Traffic Condition (Color)	Speed Interval	Description
Black	0	Indicates extremely slow or stopped traffic.
Red	<20 km/h	Highway traffic is moving slow and could indicate an accident or traffic jam on that route.
Orange	>20 km/h and <40 km/h	Indicate medium amount of traffic.
Green	>40 km/h	Indicate that traffic is fast.

**Table 3:** Distribution of the 212 measurements by the possible states of each categorical variable.

	Position of Window		Presence of Rain		Traffic Condition			
**Categories**	**Open**	**Closed**	**Yes**	**No**	**Very Slow**	**Slow**	**Medium**	**Fast**
No. of samples	95	117	18	194	48	55	58	51
Encoding	1	0	1	0	0	1	2	3

**Table 4:** Model coefficients (with confidence interval in parenthesis) and goodness of fit metrics for traffic vs. power model.

Linear Regression Coefficients		Goodness of Fit			
$a_{0}$	$a_{1}$	**MSE**	**R-Squared**	**F-Statistic**	**Prob (F)**
4.6124 (4.342–4.883)	0.0787 (0.072–0.085)	0.3442	0.72	539.7	6.04 × 10^−60^

**Table 5:** Model coefficients (with confidence interval in parenthesis) and goodness of fit metrics for rain vs. power linear model.

Linear Regression Coefficients		Goodness of Fit			
$b_{0}$	$b_{1}$	**MSE**	**R-Squared**	**F-Statistic**	**Prob (F)**
0.2278 (0.100–0.355)	0.0037 (0.001–0.007)	0.07649	0.025	5.346	0.0217

**Table 6:** Model coefficients (with confidence interval in parenthesis) and goodness of fit metrics for rain vs. power logistic model.

Logistic Regression Coefficients		Goodness of Fit
$b_{0}$	$b_{1}$	**Pseudo R-Squared**
−0.5264 (−2.100 1.047)	0.0516 (0.007–0.097)	0.04482

**Table 7:** Model coefficients (with confidence interval in parenthesis) and goodness of fit metrics for rain vs. power linear model.

Linear Regression Coefficients		Goodness of Fit			
$c_{0}$	$c_{1}$	**MSE**	**R-Squared**	**F-Statistic**	**Prob (F)**
0.6718 (0.444–0.900)	0.0058 (0.000–0.011)	0.2449	0.019	4.091	0.0444

**Table 8:** Model coefficients (with confidence interval in parenthesis) and goodness of fit metrics for rain vs. power logistic model.

Logistic Regression Coefficients		Goodness of Fit
$c_{0}$	$c_{1}$	**Pseudo R-squared**
0.7088 (−0.228 1.645)	0.0238 (0.000–0.047)	0.01403

**Table 9:** Model coefficients (with confidence interval in parenthesis) and goodness of fit metrics for speed vs. power model.

Linear Regression Coefficients		Goodness of Fit			
$d_{0}$	$d_{1}$	**MSE**	**R-Squared**	**F-Statistic**	**Prob (F)**
100.5496 (93.70–107.399)	1.8095 (1.640–1.979)	220.8105	0.68	445.2	8.60 × 10^−54^

**Table 10:** Model coefficients and goodness of fit metrics for the speed vs. all other variables model.

Linear Regression Coefficients							Goodness of Fit			
$e_{0}$	$e_{1}$	$e_{2}$	$e_{3}$	$e_{4}$	$e_{5}$	$e_{6}$	**MSE**	**R-Squared**	**F-Statistic**	**Prob (F)**
14.19	0.2544	11.21	30.91	60.50	−2.70	−1.08	48.80	0.931	460.04	5.50 × 10^−116^

## Data Availability

The data collected and presented in this paper is avaiable in [44].
